# Supplementary material for: Content-rich biological network constructed by mining PubMed abstracts
Source: BMC Bioinformatics. 2004 Oct 8;5:147. doi: 10.1186/1471-2105-5-147 (PMC528731; doi:10.1186/1471-2105-5-147)
Supplement: Additional File 5 — The original Chilibot query results of the term "long-term potentiation (LTP)" and 22 other terms, limiting the latest references analyzed to the years 1990, 1995, 2000, and 2004. [file 1471-2105-5-147-S5.bz2 › chilibotAdditionalFile5/ltp2004/html/PKA_ARC.html]

 


 **PKA** and **ARC** 
  
Found 9 abstracts in PubMed,  **9 abstracts were retrieved and analyzed**.  


---

 Search Google  |
 PDF files only 
|  EDU domain only 

---

**Interactive relationship** (e.g. stimulation, inhibition, etc)

- Therefore, increased  **PKA**  activity maintains cellular tolerance to mu opioid receptor agonists in  **ARC**  neurosecretory cells caused by chronic morphine treatment.  Ref: 9618432 J Pharmacol Exp Ther, 1998
- This induction requires the activity of  **PKA**  and mitogen activated protein kinase, suggesting a neuron specific pathway for the activation of arg3.1  **arc**  expression.  Ref: 11466419 J Neurosci, 2001

- :-)

  **Parallel relationship** (e.g. studied together, co-existance, homology, etc.)

  - The present study examined protein kinase A  **PKA**  and protein kinase C PKC involvement in the maintenance of cellular tolerance to mu opioid receptor agonists resulting from chronic opiate exposure in neurosecretory cells of the hypothalamic arcuate nucleus  **ARC** .  Ref: 9618432 J Pharmacol Exp Ther, 1998
  - A fluorescently labeled adenosine oligoarginine conjugate  **ARC** , nanomolar bisubstrate analogue type inhibitor of basophilic protein kinases  **PKA**  and PKC, readily enters cells of different origin and localizes into cytoplasm and nucleus.  Ref: 12127517 Bioorg Med Chem Lett, 2002
  - These results demonstrate that ghrelin directly interacts with NPY neurons in the  **ARC**  to induce calcium signaling via  **PKA**  and N type calcium channel dependent mechanisms.  Ref: 12663466 Diabetes, 2003
  - When an aqueous anaerobic solution pH 5 or 9 of PSH  **pKa**  = 7.7 and either 2 methyl 2 nitrosopropane MNP or nitromethane NM were irradiated lambda > 300 nm with a Xe  **arc**  lamp, the corresponding purine 6 thiyl PS. radical adduct and the reduced form of the spin trap MNP H. or CH3NO2.  Ref: 7870762 Photochem Photobiol, 1994
